# Disproportionate Impact of Sexually Transmitted Diseases on Women^1^

**DOI:** 10.3201/eid1011.040623_02

**Published:** 2004-11

**Authors:** Sevgi O. Aral, Sarah Hawkes, Ann Biddlecom, Nancy Padian

**Affiliations:** * Centers for Disease Control and Prevention, Atlanta, Georgia, USA;; † London School of Hygiene and Tropical Medicine, London, United Kingdom;; ‡ Alan Guttmacher Institute, New York City, New York, USA;; § University of California at San Francisco, San Francisco, California, USA

**Keywords:** gender differentials, vulnerability, STD/HIV prevention

Worldwide, sexually transmitted diseases (STDs) and HIV affect women more than men. This gender differential is greater in developing countries than in industrialized countries, and biological, social, cultural, and economic factors all contribute to the gender differential in STD/HIV. Larger mucosal surface area, microlesions caused during sex (particularly forced sex), and the presence of more HIV in semen than in vaginal secretions all contribute to women's greater vulnerability to STDs and HIV.

Their sex partners' behaviors also put women at risk for STDs and HIV. Culturally, men are expected to have multiple sex partners, including sex workers, and women may risk abuse or suspicion of infidelity if they refuse sex or request protection. Financial and material dependence on men renders women economically more vulnerable to STDs and HIV. Often women are under pressure to find a husband or bring home money, which in the absence of viable alternatives leads them into sex work. Effective prevention of STDs and HIV necessitates large-scale social, cultural, and economic changes and female-controlled prevention, such as microbicides.

